# Epoxy-Oxylipins and Soluble Epoxide Hydrolase Metabolic Pathway as Targets for NSAID-Induced Gastroenteropathy and Inflammation-Associated Carcinogenesis

**DOI:** 10.3389/fphar.2019.00731

**Published:** 2019-06-25

**Authors:** Ryan D. Jones, Jie Liao, Xin Tong, Dandan Xu, Leyu Sun, Haonan Li, Guang-Yu Yang

**Affiliations:** Department of Pathology, Feinberg School of Medicine, Northwestern University, Chicago, IL, United States

**Keywords:** oxylipin, soluble epoxide hydrolase, non-steroidal anti-inflammatory drug, inflammation, carcinogenesis

## Abstract

Polyunsaturated fatty acids (PUFAs) including epoxide-modified ω-3 and ω-6 fatty acids are made *via* oxidation to create highly polarized carbon-oxygen bonds crucial to their function as signaling molecules. A critical PUFA, arachidonic acid (ARA), is metabolized to a diverse set of lipids signaling molecules through cyclooxygenase (COX), lipoxygenase (LOX), cytochrome P450 epoxygenase, or cytochrome P450 hydroxylase; however, the majority of ARA is metabolized into anti-inflammatory epoxides *via* cytochrome P450 enzymes. These short-lived epoxide lipids are rapidly metabolized or inactivated by the soluble epoxide hydrolase (sEH) into diol-containing products. sEH inhibition or knockout has been a practical approach to study the biology of the epoxide lipids, and has been shown to effectively treat inflammatory conditions in the preclinical models including gastrointestinal ulcers and colitis by shifting oxylipins to epoxide profiles, inhibiting inflammatory cell infiltration and activation, and enhancing epithelial cell defense *via* increased mucin production, thus providing further evidence for the role of sEH as a pro-inflammatory protein. Non-steroidal anti-inflammatory drugs (NSAIDs) with COX-inhibitor activity are among the most commonly used analgesics and have demonstrated applications in the management of cardiovascular disease and intriguingly cancer. Major side effects of NSAIDs however are gastrointestinal ulcers which frequently precludes their long-term application. In this review, we hope to bridge the gap between NSAID toxicity and sEH-mediated metabolic pathways to focus on the role of epoxy fatty acid metabolic pathway of PUFAs in NSAIDS-ulcer formation and healing as well as inflammation-related carcinogenesis. Specifically we address the potential application of sEH inhibition to enhance ulcer healing at the site of inflammation *via* their activity on altered lipid signaling, mitochondrial function, and diminished reactive oxygen species, and further discuss the significance of dual COX and sEH inhibitor in anti-inflammation and carcinogenesis.

## Introduction

Inflammation is a diverse and complex series of pathogenetic processes, which act to protect host organisms against infectious pathogens and damaged cells or tissues. Inflammation is initiated *via* cellular and molecular signaling, and it is necessary for clearance of infections and tissue damage and for tissue repair. On the other hand, the inflammatory process itself can cause significant harm to the host (Jaeschke and Smith, 1997). One such family of molecular mediators or signaling of inflammation is arachidonic acid (ARA) and its metabolites.

**Figure 1:**
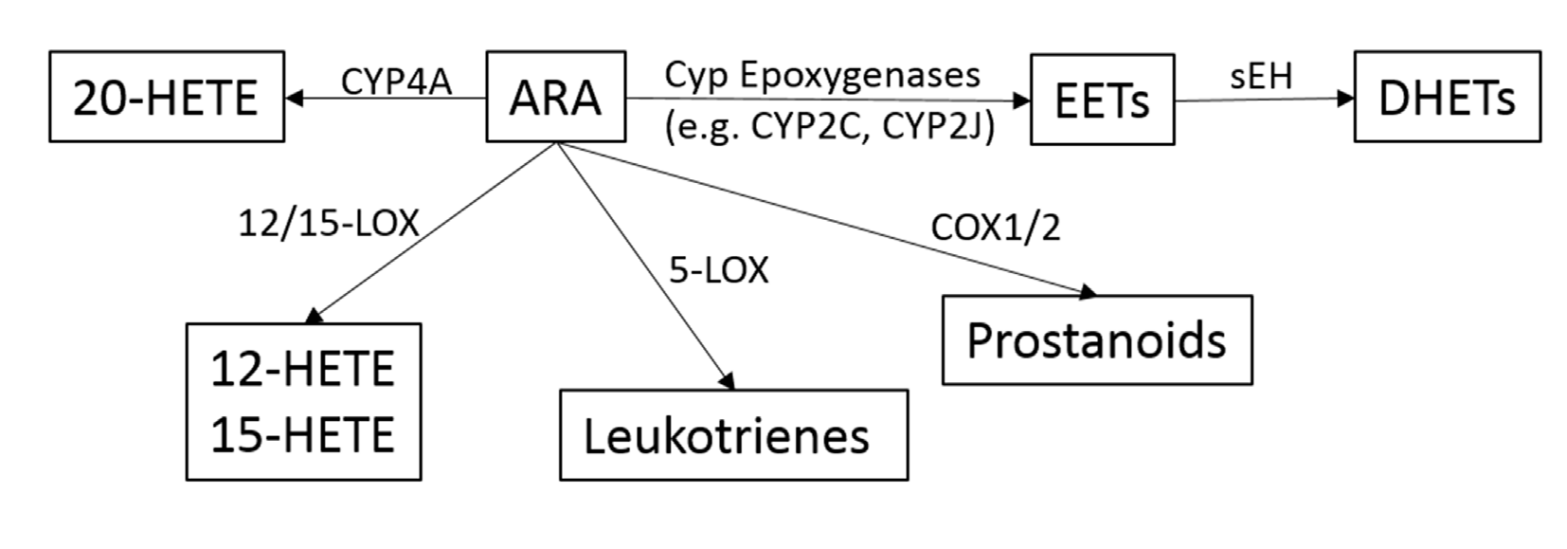
Overview of the metabolic pathways of the arachidonic acid (ARA) cascade.

ARA is a pivotal molecule in inflammation, which when released in response to tissue injury can be metabolized into three broad pathways governed by cyclooxygenase (COX), lipoxygenase (LOX), and cytochrome P450 enzymes (including epoxygenase and hydroxylase) (as outlined in **Figure 1**) (Morisseau and Hammock, 2012). Downstream active molecules from ARA metabolism include prostaglandins (PGs), leukotrienes, epoxyeicosanoids, and hydroxyeicosatetraenoic acid. In addition to ARA, other polyunsaturated fatty acids (PUFAs) including eicosapentaenoic acid (EPA) and docosahexaenoic acid (DHA) are also substrates for these same enzymes (Hiesinger et al., 2019). Of particular interest are the epoxyeicosatrienoic acids (EETs), the metabolites of cytochrome p450 epoxygenase. EETs play important roles in gastrointestinal (GI) epithelial integrity and wound healing, and are key negative regulators of inflammation (Zhang et al., 2012; Zhang et al., 2013b; Zhang et al., 2013c). The effects of EETs have been extensively reviewed by Spector and Kim (2015).

EETs are substrates for the enzyme soluble epoxide hydrolase (sEH), which rapidly converts EETs to dihydroxyeicosatrienoic acids (DHETs) (Capdevila et al., 2000; Spector et al., 2004). The epoxide fatty acids generated from EPA and ARA are also physiologically active, and are substrates of sEH (Morisseau and Hammock, 2012). These diol-containing DHETs have drastically reduced biologic activity. Thus, inhibition of sEH has been extensively studied as a mechanism to increase the longevity of anti-inflammatory EETs *via* the discovery of sEH inhibitors, particularly in combination with other inhibitors as multi-target therapies, which were recently reviewed (Hiesinger et al., 2019). sEH inhibitors have been shown to robustly decrease sEH activity with little to no toxicity in animal models. Further, these compounds have proven effective to 1) decrease GI ulcers induced by non-steroidal anti-inflammatory drugs (NSAIDs) (Goswami et al., 2016; Goswami et al., 2017; Yang, 2018), 2) prevent carcinogenesis in murine models of colorectal and pancreatic tumors (Liao et al., 2016a; Liao et al., 2016b; Wang et al., 2018b; Yang, 2018), and 3) decrease chronic inflammation in mouse models for both colitis and pancreatitis (Zhang et al., 2012; Zhang et al., 2013b; Zhang et al., 2013c; Goswami et al., 2016; Liao et al., 2016a; Goswami et al., 2017; Wang et al., 2018b; Yang, 2018).

Prostaglandins (PGs), produced through oxygenation of ARA *via* COX enzymes, result in a diverse family of structures that modulate many functions including vascular tone, platelet aggregation, and inflammation (Ricciotti and FitzGerald, 2011). The NSAID family of drugs act through inhibition of COX-1 and/or COX-2. Specific inhibitors against COX enzymes have also been developed. In addition to NSAIDs anti-inflammatory properties, they have been shown to decrease the risk of several cancers including colorectal adenocarcinomas (Cao et al., 2016; Chan and Ladabaum, 2016; Tsoi et al., 2018). Long-term NSAID use, however, often leads to severe GI tract ulcers and potentially life-threatening bleeding precluding their widespread use in chemoprevention (Sostres et al., 2010).

This review aims to highlight a potential strategy combining sEH and COX inhibition for chemoprevention and inflammatory conditions while also mitigating the adverse side effects of single agent sEH inhibitors and NSAIDs.

## Polyunsaturated Fatty Acid Metabolism and EETs

The 20-carbon ω-6 PUFA ARA is a lipid signaling molecule that resides among phospholipids and is a key substrate for a variety of downstream cell signaling mediators known as eicosanoids. ARA is broadly metabolized into one of three main pathways: 1) by COX into prostaglandins; 2) by 5-LOX into leukotrienes; and 3) by cytochrome P450 (CYP 450) epoxygenase and hydroxylase to form epoxyeicosanoids and hydroxyeicosatetraenoic acid. The EETs are signaling molecules formed within various types of cells by the metabolism of ARA (Capdevila et al., 2000; Capdevila and Falck, 2001). Several CYP 450 enzymes are involved in EET production, and include CYP2C and CYP2J, which epoxidize ARA at one of the four double bonds resulting in two different enantiomers for each EET regioisomer (Capdevila et al., 2000).

EETs can act as both autocrine and paracrine mediators of inflammation, angiogenesis, apoptosis, fibrinolysis, mitogenesis, vasodilation, and bronchodilation (Karara et al., 1990; Karara et al., 1991; Karara et al., 1992; Weintraub et al., 1999). The mechanism of their activity is not fully known, but likely involves two possible mechanisms. One postulated mechanism is that EETs function through membrane receptors such as G protein-coupled receptors or tyrosine kinase receptors to activate intracellular signaling cascades (Li and Campbell, 1997; Hoebel and Graier, 1998; Chen et al., 2000; Fukao et al., 2001; Node et al., 2001; Seubert et al., 2004; Wang et al., 2005). The other possible mechanism is that EETs directly interact with transcriptions factors, ion channels, or intermediary signal transduction proteins. But, the definitive EET-binding receptor/s or signaling proteins are not yet identified.

The catabolism of EETs can occur through several pathways including the main pathway of epoxide hydrolysis by sEH into DHETs, but can also occur through β-oxidation (Fang et al., 2000), fatty acid chain elongation (Fang et al., 1995; Fang et al., 1996; Fang et al., 2001), and rarely through CYP 450 ω-oxidases (Cowart et al., 2002), COX (Zhang et al., 1992; Carroll et al., 1993), LOX (Pace-Asciak et al., 1983), and glutathione-S-transferase (Spearman et al., 1985). Recently, COX-derived proangiogenic metabolites of EETs were identified (Rand et al., 2017). EETs have much higher anti-inflammatory and promoting tissue repair activities compared with DHETs produced by sEH, providing a rationale for sEH inhibition (Inceoglu et al., 2007).

## Non-Steroidal Anti-Inflammatory Drugs, Side Effects, and Their Role in Chemoprevention

Acetylsalicylic acid (aspirin) was the first NSAID synthesized and developed for commercial use in 1897 by Felix Hoffman, and it became widely used for its analgesic and anti-inflammatory effects (Sostres et al., 2010). It took approximately 40 years before it was definitively linked to GI injury. It was not until the 1970s that it was discovered that aspirin acts to inhibit prostaglandin production. Today, over 20 NSAIDs are commercially available with varied toxicities and pharmacokinetics and include non-selective COX-1/COX-2 inhibitors (e.g., aspirin, ibuprofen, naproxen, diclofenac) and COX-2 selective inhibitors (coxibs, e.g., celecoxib).

NSAIDs are among the most commonly administered drugs worldwide, as they are available by prescription and over-the-counter. Excitingly, they are also among the most promising agents for prevention of cardiovascular diseases and cancer including colorectal cancer (CRC) (Fischer et al., 2011; Wongrakpanich et al., 2018). One of the major adverse effects of NSAIDs therapy is GI injury, including ulceration, bleeding, inflammation, and even perforation (Wolfe and Singh, 1999; Sudano et al., 2012; Tsoi, 2018). This is a serious clinical challenge causing a major burden on the health care system (specifically, in the US, >100,000 patients are hospitalized with serious NSAID-related GI complications annually, including 16,500 deaths) (Wolfe and Singh, 1999; Bjarnason et al., 2018). In addition to NSAIDs toxicity in the stomach, novel imaging techniques (e.g., capsule endoscopy) have identified the small intestine as a major target organ of toxicity, and lower GI bleeding events may even appear more frequently than upper GI events (Maiden, 2009; Sostres and Lanas, 2011). The local topical effect of NSAIDs is partially responsible for the GI-tract ulceration and bleeding, as co-prescription of aspirin and proton pump inhibitors (PPIs) or enteric-coating of NSAIDs both provide some protection against NSAID-induced gastritis and gastric ulcers; however, these treatment strategies actually increase the incidence of small-bowel ulcers. The non-topical systemic effect of NSAID use appears to be key, as enteric coating, parenteral, and rectal administration all continue to result in gastroenteropathies (Sostres et al., 2010). At least two-thirds of both long-term (>3 months) and short-term (>1 week) NSAID/coxibs users exhibit ulcers in the jejunum and ileum (Maiden, 2009). To date there is no therapy or preventive treatment for NSAIDs-induced GI ulcers available.

In contrast to the adverse ulcer-causing effects, several clinical trials and epidemiological and animal studies have clearly demonstrated that aspirin or NSAIDs actually decrease the risk and mortality of CRC and prevent colon adenoma formation (Dubois, 2009; Burn et al., 2011; Fischer et al., 2011; Cao et al., 2016). To acknowledge this supporting evidence, in 2016 the U.S. Preventive Services Task Force (USPSTF) recommended the use of aspirin to prevent CRC and cardiovascular disease for adults aged between 50 and 70 in the U.S. This recommendation distinguishes aspirin as the first pharmacologic agent to be endorsed by the USPSTF for chemoprevention of a cancer in a population not characterized as high risk (Cao et al., 2016; Chan and Ladabaum, 2016). In addition, notable chemopreventive clinical trials studied the use of aspirin for the prevention of adenomas and carcinomas in patients with Lynch syndrome (Burn et al., 2011) and the use of celecoxib for the reduction of sporadic colorectal adenoma (Dubois, 2009). To achieve significant chemoprevention while also reducing the adverse effects of GI ulcers, alternative approaches with either low doses of NSAIDs or in combination with other chemopreventive agents have been tried with little success (Fischer et al., 2011).

## Soluble Epoxide Hydrolase

Epoxides are cyclic three atom rings which are highly reactive and are metabolized by epoxide hydrolases by adding water forming a diol group in a dihydroxylation reaction. The epoxide hydrolase family is comprised of numerous enzymes including: microsomal epoxide hydrolase (mEH), sEH, leukotriene A_4_ hydrolase, cholesterol 5,6-oxide hydrolase, and epoxide hydrolase 3 (Fretland and Omiecinski, 2000; Decker et al., 2012). The major epoxide hydrolase involved in EET metabolism is sEH, encoded by the EPHX-2 gene located on chromosome 8 (Larsson et al., 1995; Sandberg and Meijer, 1996). The 60-kDa sEH forms a homodimer with each monomer containing N-terminal domain lipid phosphatase activity with unknown physiologic significance, as well as a functionally independent C-terminal domain epoxide hydrolase activity (Argiriadi et al., 1999; Cronin et al., 2003; Newman et al., 2003).

In the early 1990s, concerns about the potential adverse effects of a chronic loss of sEH have centered around the clearance of xenobiotics, particularly the potential loss of function to clear epoxide-containing carcinogens. These concerns have been well studied and demonstrated that these xenobiotics are not the substrates of sEH (Seidegard and Ekstrom, 1997; Fretland and Omiecinski, 2000; Morisseau and Hammock, 2005; Shimada, 2006; Morisseau and Hammock, 2007; Decker et al., 2009). Studies of evolution of the microsomal EH (mEH) and sEH show that although they have common origins, they diverge at the level of prokaryotes and their substrate selectivity is vastly different (Fretland and Omiecinski, 2000). mEH rapidly degrades carcinogenic epoxides on cyclic systems (e.g., aflatoxin epoxide and benzo[a]pyrene epoxide) (Shimada, 2006). To date, sEH has not been shown to metabolize a single carcinogenic epoxide and endogenous eicosanoids are identified as its only substrates (Seidegard and Ekstrom, 1997; Decker et al., 2009). sEH inhibitors have been cross-tested on the mEH, which show a much greater selectivity for sEH (Morisseau and Hammock, 2005; Morisseau and Hammock, 2007). Most importantly, the fatty epoxides stabilized through sEH inhibition display a strong anti-inflammatory action (Norwood et al., 2010).

sEH shows high tissue expression in liver, intestine, kidneys, brain, and vasculature, with lower expression in spleen, lung, and testes (VanRollins et al., 1993; Fang et al., 1995; Johansson et al., 1995; Yu et al., 2000). The subcellular localization is variable; however, sEH is mostly localized to the cytosol with peroxisomal involvement in hepatocytes and renal proximal tubule epithelial cells (Hollinshead and Meijer, 1988; Eriksson et al., 1991; Mullen et al., 1999; Enayetallah et al., 2006). The biological activity and significance of different cell types and different subcellular localization of sEH have not been fully elucidated.

## Development and Application of Selective sEH Inhibitors

Inhibitors have been developed for sEH, which have been instrumental in understanding the biology of EETs and sEH, as well as for testing their use in practical applications of disease. Importantly, through decades of studies, inhibitors have been selected for their stability, high selectivity against sEH, and high potency. The first iterations of sEH inhibitors were epoxide-containing *trans*-3-phenylglycidol and chalcone oxide molecules, and while these inhibitors are selective and potent, they are unstable with only temporary effects on sEH (Mullin and Hammock, 1982; Dietze et al., 1991; Dietze et al., 1993; Morisseau et al., 1998).

Newer classes of sEH inhibitors include urea- and carbamate-based molecules, which are more stable than previous inhibitors, and are also highly selective and potent (Morisseau et al., 1999). One inhibitor in this class is *N,N’*-dicyclohexylurea (DCU). DCU is the basis for several urea-based inhibitors with increased water solubility, and which bind to the active site of sEH *via* the urea subgroup with hydrogen bonds and salt bridges (Argiriadi et al., 2000; Morisseau et al., 2002; Kim et al., 2004).

The most recent and well-studied sEH inhibitors are 12-(3-adamantyl-ureido)-dodecanoic acid (AUDA) compounds. These AUDA compounds have been modified to bind the catalytic site of sEH *via* hydrogen bonds and have physical properties ideal for pharmacologic use including high water solubility, oral bioavailability, low melting point, increased stability, and high potency. Trans-4-[4-(3-adamantan-1-ylureido)cyclohexyl oxy]benzoic acid (t-AUCB) is another highly promising sEH inhibitor. Thus far, sEH inhibitors have been demonstrated to be safe, with very few side effects. The effective dosages show very little toxicity.

## sEH Inhibition Increases EETs, Promotes Ulcer Healing, and Inhibits Pancreatitis, Colitis, and Inflammation-Associated Cancer in Mice

Chronic inflammation has been demonstrated to be among the most important factors leading to carcinogenesis. Obesity, inflammatory bowel disease (IBD), and chronic colitis and pancreatitis are some specific examples of inflammatory conditions that are known risk factors for cancer with shared mechanisms involving altered epoxide fatty acid metabolites and sEH (Zhang et al., 2013; Liao et al., 2016a; Wang et al., 2018b; Yang, 2018). As a result of chronic inflammation there is overproduction of reactive oxygen and nitrogen species (RONS), as well as dysregulation of ARA metabolites. Inflammatory cell infiltration driven by neutrophils, macrophages, and subsequently lymphocytes alters tissue microenvironments with an enhanced production of RONS (e.g., superoxide) and ARA metabolites (e.g., PGE2 and LTB4). Leukocytes also act to modify vasculature through effects on endothelial cells (Yang et al., 2009).

Mounting evidence links EETs and sEH inhibition to treatment for chronic inflammatory conditions and inflammation-associated cancer. First, sEH has been shown to be overexpressed in human colitis and CRC (Zhang et al., 2013b). Second, recent studies demonstrated that sEH bridges obesity to colonic inflammation and potentially to colorectal carcinogenesis (Wang et al., 2018b; Yang, 2018; Yang et al., 2018). And finally, sEH inhibition blocks colitis and its associated carcinogenesis (Zhang et al., 2012; Zhang et al., 2013b; Zhang et al., 2013c) and NSAID-induced GI ulcers (Goswami et al., 2016; Goswami et al., 2017). However, the relative contribution and mechanism of sEH in sporadic colon carcinogenesis is not fully known.

Initial studies regarding the anti-inflammatory effects of EETs showed that physiologic concentration of EETs or overexpression of CYP2J2 decreases cytokine-mediated endothelial cell adhesion molecule expression, thus preventing leukocyte recruitment *via* a mechanism involving NF-κB (Node et al., 1999). Inhibition of EET catabolism through sEH inhibitors results in a significant increase in their anti-inflammatory properties, and effectively treat mouse models of inflammatory diseases.

EETs cause an increased activity and expression of peroxisome proliferator-activated receptor-gamma (PPAR-γ), a low-affinity nuclear hormone receptor involved in fatty acid metabolism, and inhibition of the NF-κB pathway (Stienstra et al., 2007; Ward and Tan, 2007). It is likely that at least a portion of the anti-inflammatory effects of EETs act through PPAR-γ as cell culture models show that inhibition of this nuclear hormone receptor diminishes this effect. The role of PPAR-γ in inflammation is through inhibition of activity of pro-inflammatory transcription factors activator protein-1 (AP-1), signal transducers and activators of transcription (STAT), and nuclear factor κB (NF-κB). Thus, in addition to increasing EETs, ligand activation of PPAR-γ is another promising strategy for anti-inflammatory activity (Dubuquoy et al., 2006).

### sEH Inhibition Can Block NSAID-Induced Gastrointestinal Ulcers

The underlying mechanisms of NSAID-induced GI ulcers are not fully understood; however, they likely involve endoplasmic reticulum (ER)/mitochondrial stress and mucinous epithelia-microvascular barrier breakdown (Hawkey, 1996; Wolfe and Singh, 1999; Cheng et al., 2015). The effects of NSAIDs/aspirin on enterocytes and on vascular endothelial cells in the lamina propria are considered toxic/pathogenic based to the findings of i) the relatively high concentrations (mM) of conjugated NSAIDs through the enterohepatic circulation (Seitz and Boelsterli, 1998; Treinen-Moslen and Kanz, 2006), ii) direct toxic effects on intestinal mucosal epithelial and endothelial cells (break down the mucosal-vascular barrier), iii) effects on mitochondria (Somasundaram et al., 1997) and ER stress (Tsutsumi et al., 2004), and iv) mucosal injury related to active inflammation.

**Figure 2:**
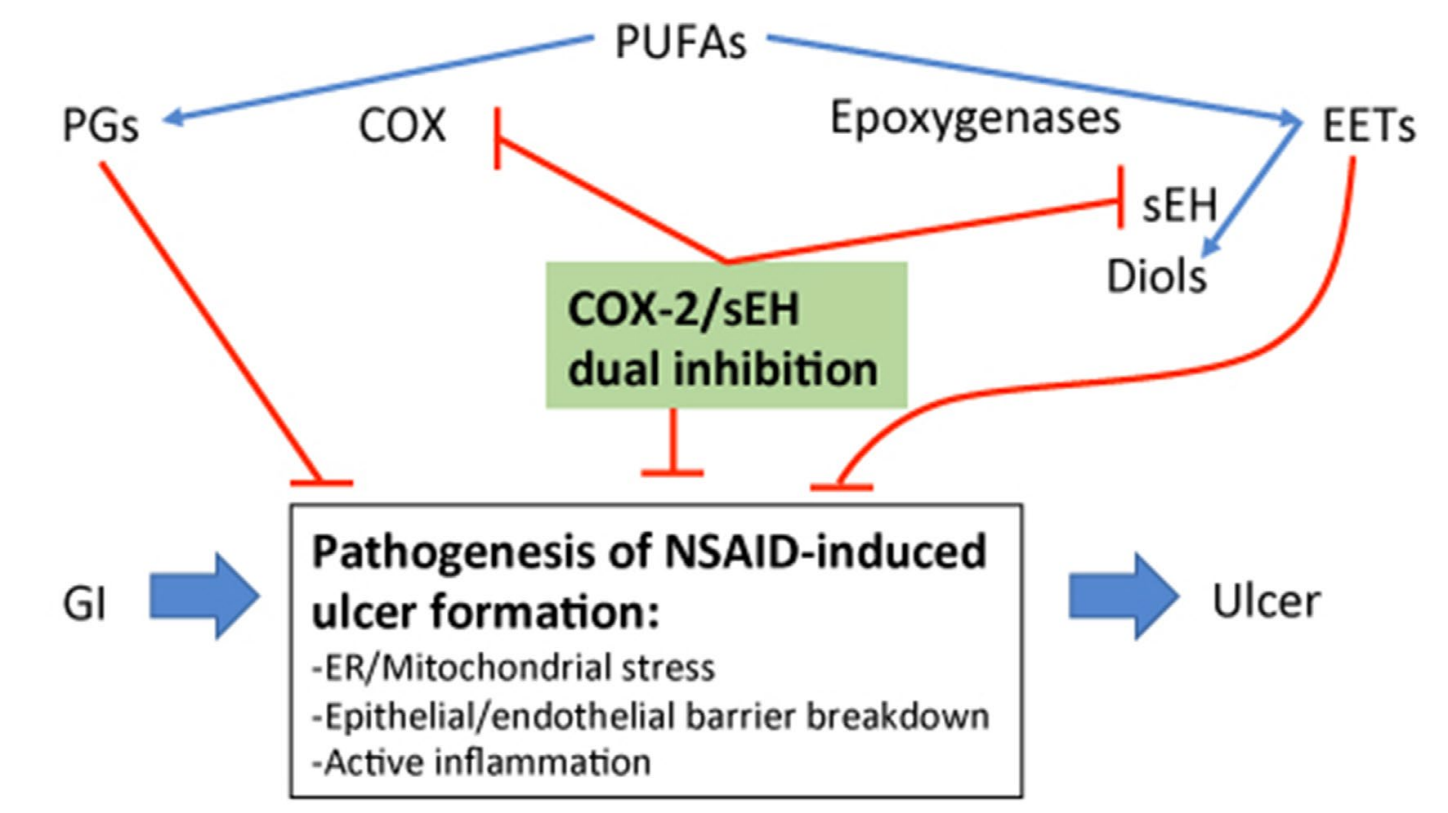
Outline of the potential mechanism of sEH inhibition-stabilized EETs in blocking NSAID-induced GI ulcers.

NSAID-induced GI ulcers can be counter-balanced by epoxides through direct administration of EETs or indirectly by increasing EETs through sEH inhibition (Goswami et al., 2016; Goswami et al., 2017; Yang, 2018). sEH inhibition increases EET bioavailability and yields significant anti-inflammatory effects (Node et al., 1999; Schmelzer et al., 2005; Imig and Hammock, 2009; Norwood et al., 2010). In addition to increasing EET availability, a partial mechanism for the anti-inflammatory effect of sEH inhibition is through a decrease of its product DHET, which has been shown to be important for monocyte chemoattractant protein-1 (MCP-1)-mediated monocyte chemotaxis (Kundu et al., 2013). Thus, co-administration of NSAIDs and a sEH inhibitor has potential synergistic effects of modulating oxylipin profile toward the resolution of inflammation (Schmelzer et al., 2006; Zhang et al., 2014a; Zhang et al., 2014b). The potential mechanistic role of sEH inhibition against NSAID-induced GI ulcers is likely either through decreasing monocyte recruitment and inflammation (Rainsford, 1993; Kundu et al., 2013), blocking NSAID-induced ER/mitochondrial stress (Hawkey, 1996; Inceoglu et al., 2017) to reduce epithelial-vascular barrier injury (Rainsford, 1993), or through enhancing the tissue repair process and angiogenesis (Panigrahy et al., 2013) as outlined in **Figure 2**.

### Chronic Pancreatitis and Carcinogenesis

One of the most common conditions leading to pancreatic tumorigenesis is the inflammatory condition chronic pancreatitis. In the development of chronic pancreatitis aberrant ARA metabolism partially mediated by sEH acts to promote inflammation with a decrease of the anti-inflammatory EETs. Inhibition of sEH has been demonstrated as a successful strategy in murine models to inhibit chronic pancreatitis and the development of pancreatic intraepithelial neoplasms (PanIN) using trans-4-{4-[3-(4-chloro-3-trifluoromethyl-phenyl)-ureido]-cyclohexyloxy}-pyridine-2-carboxylic acid methylamide (t-CUPM) in KRAS-mutant mice (Liao et al., 2016a; Liao et al., 2016b). t-CUPM is a novel molecule with dual inhibition of sEH and RAF1 proto-oncogene serine/threonine kinase (c-RAF). This effect was due to both the anti-inflammatory effects of sEH inhibition and the blockade of constitutive KRAS signaling through c-RAF inhibition.

### Inflammatory Bowel Disease, Colorectal Carcinogenesis, and the Role of sEH and COX2

A well-recognized risk factor for sporadic colon cancer is chronic inflammation, in which aberrant PUFA metabolism, particularly ARA, is believed to be the key inflammatory mediator contributing to carcinogenesis (Ding et al., 2003; Hermanova et al., 2008). Using IBD as an example model, studies have shown that increased prostaglandins, prostaglandin synthase activity, and mucosal expression of COX-2 all correlate with the severity of IBD activity. NSAIDs are especially promising in chemoprevention for IBD, where there is a direct relationship between inflammation and carcinogenesis. Unfortunately, the clinical application of NSAIDs with COX inhibitory activity tends to exacerbate IBD. These findings are likely multifactorial related to both delayed ulcer healing, a known side effect of NSAIDs, as well as a shunt of ARA substrates to the LOX pathway leading to overproduction of the inflammatory 5-LOX-LTB_4_ pathway (Goswami et al., 2016).

Extensive epidemiological and experimental reports show COX-2 has a prominent role in carcinogenesis. Much of these observations come from the effects of aspirin and other NSAIDs resulting in the decreased rate of CRCs, including notable chemopreventive clinical trials testing the use of aspirin in the prevention of GI adenomas and carcinomas in patients with Lynch syndrome (Burn et al., 2011), and celecoxib for the reduction of colon adenomas (Dubois, 2009). COX-2 has been shown to be overexpressed by colon tumors, and COX-2 deficient mice show a decrease in APC-associated carcinogenesis. Further, under the inflammatory conditions of ulcerative colitis (UC), COX-2 is commonly overexpressed in the sequence of UC progression to carcinoma, as well as in non-dysplastic epithelium. The increased expression of COX-2 in UC has been shown to be associated with carcinogenesis rather than the inflammatory activity. PGE2 production secondary to COX-2 activity promotes epithelial proliferation and crypt stem cell survival. Because of this, COX-2 selective inhibitors were developed with the hopes of anti-cancer effects without the development of GI ulcers; however, these agents showed significant cardiovascular risks in clinical trials, and one trial showed a continued side effect of GI ulcers (Dubois, 2009).

Considering that sEH is overexpressed in human colitis and CRC (Zhang et al., 2013b), and that sEH inhibition blocks colonic inflammation and carcinogenesis (Wang et al., 2018b; Yang, 2018; Yang et al., 2018), sEH inhibition is a promising agent for the treatment of IBD. sEH inhibition markedly inhibits the progression of inflammation and dysplasia in mouse models of UC (Yang, 1996; Yang et al., 2008; Zhang, 2012). This is in part due to the regulation of cytokines and chemokines, but they also regulate other key enzymatic pathways in ARA metabolism. By increasing EETs, there is a reduction of COX-2 and 5-LOX and their downstream metabolites leading to a reduction of symptoms associated with severe inflammation (Schmelzer et al., 2005; Zhang, 2012). sEH inhibition can be simplistically viewed as shifting ARA metabolism to an EET-rich profile and restoring an anti-inflammatory state.

## sEH inhibition—Potent Anti-Inflammatory Activity *vs* Potential Angiogenesis/Tumor Progression Action, Is It Double Edge Sword?

It has been well documented that sEH inhibition has high potential for anti-inflammatory, anti-hypertension, anti-diabetes, and anti-hyperalgesic activities. In addition to the anti-inflammatory activity, sEH inhibition is also associated with an indirect reduction of endotoxin-induced COX and LOX metabolite production *in vivo*, and EETs can transcriptionally inhibit COX-2 expression (Schmelzer et al., 2005; Wang et al., 2018a). However, one of the main concerns of sEH inhibition is related to the observations that EETs derived from the cytochrome p450 pathway actually promote angiogenesis and tumor progression (Elson and Yu, 1994; Panigrahy et al., 2012). This may be partly due to the specific fatty acids, which are epoxidized, as it appears that ω-3 epoxy fatty acid derivatives (e.g., epoxydocosapentaenoic acids) when stabilized by sEH inhibitors are anti-angiogenic and block tumor growth and metastasis, contrary to ω-6 ARA metabolites such as EETs (Zhang et al., 2013a). Furthermore, sEH inhibitors have been shown to promote tumor escape from dormancy and led to extensive metastases in multiple cancer models as a result of increased endothelium-derived EET concentration (Panigrahy et al., 2012). Another study aimed to reduce EETs through downregulation of the cytochrome p450 enzyme Cyp2c44 using PPARα agonists, and demonstrated a reduction in metastatic non-small cell lung cancer growth and angiogenesis (Skrypnyk et al., 2014). In spite of the association of EETs with angiogenesis and cancer progression, there is recent evidence that COX-derived products of EET metabolism may also contribute to angiogenesis, and the dual inhibition of sEH and COX results in a robust inhibition of tumor growth and a net negative effect on angiogenesis (Rand et al., 2017). This would further establish the COX-mediated EET metabolism as physiologically relevant and provides additional rationale for the sEH/COX dual inhibitor strategy. Two dual therapy cancer models of sEH inhibition combined with either COX-2 inhibition or c-RAF inhibition show robust tumor growth and metastasis inhibition, and in the case of the dual sEH/COX-2 inhibitor (discussed below), both bladder cancers and glioblastomas were found to be inhibited with significantly decreased angiogenesis (Liao et al., 2016b; Li et al., 2017; Wang et al., 2018a).

## Dual COX2/sEH Inhibition

To minimize the angiogenic effect of sEH inhibitor, a novel COX-2/sEH dual inhibitor has been synthesized, 4-(5-phenyl-3-{3-[3-(4-trifluoromethyl-phenyl)-ureido]-propyl}-pyrazol-1-yl)-benzenesulfonamide (PTUPB), based on the structures of celecoxib and sEH inhibitor *trans*-4-[4-(3-adamantan-1-ylureido)cyclohexyloxy]benzoic acid (*t*-AUCB) (Hwang et al., 2011), to circumvent problems associated with formulation of two drugs for co-administration (Morphy and Rankovic, 2005). Studies related to the pharmacokinetics and safety profile have demonstrated favorable characteristics (Hwang et al., 2011; Hye Khan et al., 2016). PTUPB is potent in inhibiting sEH (IC_50_, 0.9nM) and COX2 (IC_50_, 1.26uM), but not COX-1 (IC_50_, > 100uM), and is effective at suppressing inflammation (Hwang et al., 2011; Zhang et al., 2014b).

**Figure 3:**
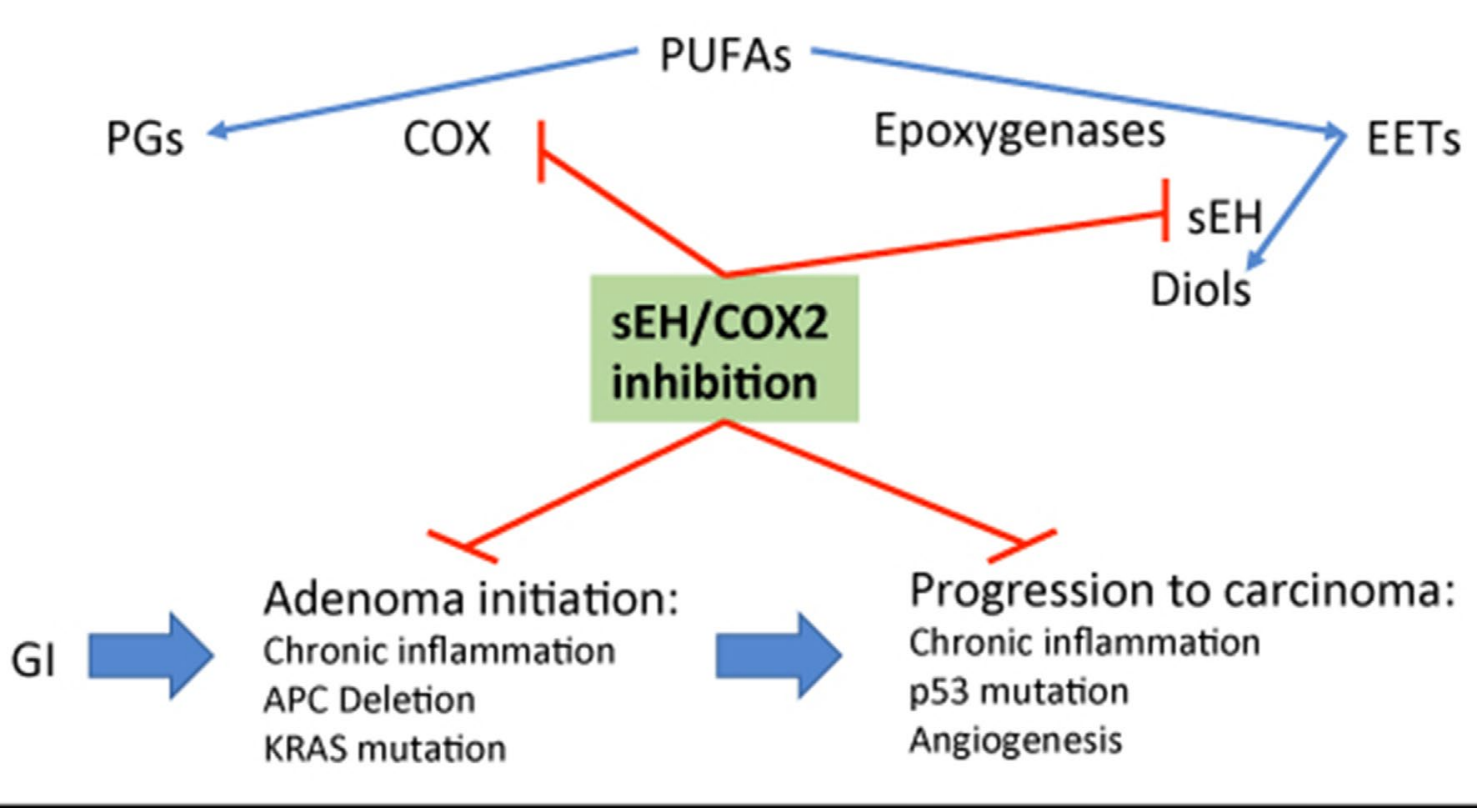
Outline of potential mechanism of sEH inhibition-stabilized EETs in inhibiting colonic adenoma—carcinoma sequence *via* targeting inflammation and its activated Wnt/mutant p53 signals.

PTUPB’s effects are more potent than celecoxib or *t*-AUCB alone and in combination (Hwang et al, 2011; Zhang et al., 2014b). PTUPB has been demonstrated to protect against metabolic abnormalities and retain kidney function in the Zucker diabetic fatty rat model (Hye Khan et al., 2016). In mouse models of cancer, PTUPB has been shown to be effective to inhibit tumor growth, metastasis, and angiogenesis, and to potentiate cisplatin-based therapies without increased toxicity (Zhang et al., 2014b; Li et al., 2017; Wang et al., 2018a). Thus, a novel strategy of co-targeting both COX2 and sEH *via* the dual inhibitor PTUPB is particularly intriguing for chemoprevention in the colorectum, especially in patients with IBD. This strategy would provide an environment enriched with EETs, with the chemopreventive benefits of COX-2 inhibition while minimizing side effects including GI tract ulceration, as outlined in **Figure 3**. Taken together, these preclinical studies highly support multi-target agents against sEH and COX2, and their potential for translating into clinical trial, and toward this direction, application for clinical trials is ongoing.

## Summary of sEH and COX2 Inhibition on Anti-Inflammatory and Carcinogenesis

The decades of studies of ARA metabolism and the sEH point to the potential of sEH inhibition and subsequent enrichment of EETs as an effective strategy for therapies in inflammatory conditions. This is particularly promising in colon and pancreas where chronic inflammatory conditions are well-documented risk factors for carcinogenesis, and therefore sEH inhibition may be ideal as a chemopreventive agent alone or in combination with other strategies. These effects have been robustly studied in mouse models, which show significant reductions in inflammation and cancer development with little to no side effects. Additionally, studies have shown that sEH inhibition can dramatically reduce the side effects of NSAIDs when given together. Thus, we believe that sEH should be further investigated in clinical trials for inflammatory conditions and inflammation-induced cancers.

One such strategy of sEH treatment involves their co-administration with NSAIDs to further modulate ARA metabolism. NSAIDs including aspirin and celecoxib are among the most widely administered medications worldwide. While they are promising for chemoprevention and in cardiovascular diseases, they also cause significant morbidity through their side effects of gastroenteropathies. These NSAID-related ulcers and bleeding are potentially life-threatening; thus, their use in chemoprevention is not practical. This emphasizes the necessity for a novel approach to harness the beneficial aspects of NSAIDs without the side effects, and one such approach may be through the dual inhibition of COX and sEH. PTUPB is one such agent, although single agent NSAIDs combined with sEH inhibitors may also be effective. This approach has the potential to revolutionize cancer treatment and prevention, and also has broad implications for the millions of people using NSAIDs chronically for pain and rheumatologic diseases.

## Author Contributions

All authors listed have made a substantial, direct, and intellectual contribution to the work and approved it for publication.

## Funding

This study was supported by NIH R01 DK10776, CA172431, and CA164041 to G-YY.

## Conflict of Interest Statement

The authors declare that the research was conducted in the absence of any commercial or financial relationships that could be construed as a potential conflict of interest.
